# The Increasing Importance of Rigorous Real-World Evidence

**DOI:** 10.1093/jncics/pkac051

**Published:** 2022-08-10

**Authors:** David D Yang, Paul L Nguyen

**Affiliations:** Department of Radiation Oncology, Dana-Farber Brigham Cancer Center, Boston, MA, USA; Harvard Radiation Oncology Program, Boston, MA, USA; Department of Radiation Oncology, Dana-Farber Brigham Cancer Center, Boston, MA, USA

Real-world evidence is derived from studies of real-world data, which are information on health care accumulated from multiple sources outside the traditional clinical research setting, including electronic health records, medical claims and billing data, product and disease registries, and personal devices and health applications (1). In contrast to traditional clinical trials, data used in real-world evidence studies are repurposed from their original intent (eg, medical claims and billing data for reimbursement) or are set up to answer a variety of research questions (eg, disease registries). Real-world evidence presents many opportunities for complementing the insights gained from traditional clinical trials given the increasing availability of real-world data with advancements in health information technology and data aggregation, the frequent lack of generalizability of traditional trials, and the increasing cost of traditional trials.

Historically, studies using real-world data have often not recapitulated the results of randomized clinical trials. For instance, a systematic comparison of population-based observational studies with randomized oncology trials found no statistically significant correlation between the hazard ratio estimates between the 2 methods (2). Multiple reasons contribute to the discrepancies observed, including differences in study populations, poor data quality, inappropriate study designs, confounders not controlled for, and other sources of bias. Although some of these reasons may reflect the reality of real-world data (namely, real-world data are often more representative of the real-world patient population compared with clinical trial populations, which must meet restrictive inclusion and exclusion criteria), these discrepancies have historically contributed to questions regarding the validity of real-world evidence (3).

With these concerns in mind, Merola et al. (4) studied the association of degarelix vs leuprolide and major adverse cardiovascular events (MACE) using a principled approach where investigators attempt to emulate or predict the results of clinical trials. This approach involves a series of prespecified checkpoints when developing the protocol, preregistration of the protocol, and additional assessment of potential sources of bias after implementation of the protocol. In the current study, the authors specifically attempted to emulate the PRONOUNCE trial, which randomly assigned men with prostate cancer and atherosclerotic cardiovascular disease to 12 months of the gonadotropin-releasing hormone (GnRH) antagonist degarelix vs the GnRH agonist leuprolide with a primary endpoint of time to first MACE (5). Enrollment into the PRONOUNCE trial was slower than projected, and the trial ultimately accrued 545 patients compared with a planned 900 participants. The investigators did not find a statistically significant difference in time to first MACE, with MACE occurring in 15 patients (5.5%) in the degarelix arm compared with 11 patients (4.1%) in the leuprolide arm (hazard ratio = 1.28, 95% confidence interval = 0.59 to 2.79, *P *=* *.53).

Patients in the study by Merola et al. (4) were identified from 3 administrative claims databases: Optum Clinformatics, IBM MarketScan, and a subset of Medicare claims data from patients with diabetes. They attempted to emulate the eligibility criteria of PRONOUNCE though could not fully apply these criteria due to lack of data on tumor staging, angiography-verified atherosclerotic cardiovascular disease, and plans for cardiac surgery at the time of treatment initiation (6). Nonetheless, after propensity score matching on more than 100 covariates, the authors found seemingly similar results for time to first MACE for degarelix, with a hazard ratio of 1.35 (95% confidence interval = 0.94 to 1.93).

A unique strength of this study is that that the authors registered their study protocol with clinicaltrials.gov on May 24, 2021, and the primary study findings were already available at that time, before the public announcement of the results of the PRONOUNCE trial on August 30, 2021. That is to say, the investigators were able to predict the observed effective size for degarelix vs leuprolide in the PRONOUNCE trial.

Several points are worth further consideration. First, the degree of similarity in the baseline patient characteristics of the current study and the PRONOUNCE trial are unknown. As the authors noted, claims codes do not identify whether a patient has angiography-verified atherosclerotic cardiovascular disease, and the distribution of cardiovascular disease severity may be different between the population of the PRONOUNCE trial compared with the current study. It is possible that the effect of degarelix vs leuprolide on MACE depends on the severity of baseline atherosclerotic cardiovascular disease. The point estimates of the hazard ratios for MACE also differed based on the data source (1.83 for Clinformatics, 1.17 for MarketScan, and 1.19 for Medicare), which may be reflective of the differences in the patient populations; however, these effect size estimates were not statistically significantly different, with *P*_homogeneity_ = .567. Despite these uncertainties, however, the investigators were able to demonstrate a similar effect size for degarelix compared with PRONOUNCE.

Second, one of the potential advantages of real-world data is that real-world data may be more representative of the overall patient population compared with traditional clinical trials, where minority populations are often underrepresented (7). Specifically, we see in the reported race data in the study by Merola et al. (4) that approximately 10.6% of the patient population is Black (assuming that patients without data on race are equally likely to be of 1 of the reported races), whereas only 5.2% of the PRONOUNCE population is Black (4,5). However, a recent study of the population-based Surveillance, Epidemiology, and End Results registry found that approximately 16.4% of patients in the United States with prostate cancer are Black (8). Thus, although real-world evidence provided by Merola et al. (4) more closely reflects the overall baseline characteristics of patients with prostate cancer in the United States, further work is necessary to ensure that patient populations of future real-world evidence studies sufficiently reflect the diversity of the patient population at large.

Additionally, it is worthwhile to contextualize the findings of this study with others that have examined the effect of GnRH agonists on MACE compared with antagonists. It has been hypothesized that GnRH agonists may lead to excess cardiovascular events due to the existence of T cells in atherosclerotic plaques that express GnRH receptors and that treatment with GnRH agonists may lead to the destabilization of vulnerable plaques (5). Multiple studies have examined the association of GnRH agonists vs antagonists and risk of cardiovascular events with mixed results (4,5,9-11). Most recently, the HERO phase III trial randomly assigned men with advanced prostate cancer to the GnRH agonist relugolix vs leuprolide and found that the 48-week cumulative incidence of MACE was statistically significant reduced from 6.2% to 2.9% (hazard ratio = 0.46). However, 2 studies using real-world data from multiple sources [including the current study by Merola et al. (4)] (11), as well as the prospective, randomized PRONOUNCE trial (5), did not demonstrate a statistically significant difference. The reasons underlying these discrepant results are unclear and deserve further investigation, though it appears that in a real-world population outside the selective context of a clinical trial, the degree to which GnRH antagonists lower the risk of MACE vs GnRH agonists in men with a history of cardiovascular disease may not be as prominent compared with results observed in the HERO trial.

Overall, the authors should be commended for carrying out this study (4). It is an excellent example of the methodologically rigorous standards that real-world evidence studies must strive for. Given the emphasis from the 21st Century Cures Act on increased use of real-world evidence to support regulatory decision-making and postmarket monitoring (12), this study highlights the increasingly prominent role that real-world evidence studies will serve.

## Funding

PLN is supported by grants from the National Cancer Institute of the National Institutes of Health under the Award Number 1R01CA240582.

## Notes

**Role of the funder:** The funder had no role in the writing of the editorial or decision to submit it for publication.

**Disclosures:** PLN receives support by grants from the National Cancer Institute of the National Institutes of Health under the Award Number 1R01CA240582 and from Bayer, Astellas, and Janssen. PLN also has consulted for Blue Earth, Janssen, Myovant, Astellas, Bayer, Cota, and Boston Scientific.

**Author contributions:** Writing-original draft, writing-review & editing: DDY and PLN.

**Disclaimers:** The authors alone are solely the responsible for the views expressed in this article and they do not represent the official views, decisions, or policy of the National Institutes of Health.

## Data Availability

No new data were generated or analyzed for this editorial.
